# Innate and adaptive immune responses against human Puumala virus infection: immunopathogenesis and suggestions for novel treatment strategies for severe hantavirus‐associated syndromes

**DOI:** 10.1111/joim.12876

**Published:** 2019-02-17

**Authors:** J. Klingström, A. Smed‐Sörensen, K. T. Maleki, C. Solà‐Riera, C. Ahlm, N. K. Björkström, H. G. Ljunggren

**Affiliations:** ^1^ Department of Medicine Huddinge Center for Infectious Medicine Karolinska Institutet Karolinska University Hospital Stockholm Sweden; ^2^ Division of Immunology and Allergy Department of Medicine Solna Karolinska Institutet Karolinska University Hospital Stockholm Sweden; ^3^ Department of Clinical Microbiology, Infectious Diseases Umeå University Hospital Umeå University Umeå Sweden

**Keywords:** hantavirus, orthohantavirus, Puumala virus, hantavirus pulmonary syndrome, haemorrhagic fever with renal syndrome, viral immunity

## Abstract

Two related hyperinflammatory syndromes are distinguished following infection of humans with hantaviruses: haemorrhagic fever with renal syndrome (HFRS) seen in Eurasia and hantavirus pulmonary syndrome (HPS) seen in the Americas. Fatality rates are high, up to 10% for HFRS and around 35%–40% for HPS. Puumala virus (PUUV) is the most common HFRS‐causing hantavirus in Europe. Here, we describe recent insights into the generation of innate and adaptive cell‐mediated immune responses following clinical infection with PUUV. First described are studies demonstrating a marked redistribution of peripheral blood mononuclear phagocytes (MNP) to the airways, a process that may underlie local immune activation at the site of primary infection. We then describe observations of an excessive natural killer (NK) cell activation and the persistence of highly elevated numbers of NK cells in peripheral blood following PUUV infection. A similar vigorous CD8 Tcell response is also described, though Tcell responses decline with viraemia. Like MNPs, many NK cells and CD8 T cells also localize to the lung upon acute PUUV infection. Following this, findings demonstrating the ability of hantaviruses, including PUUV, to cause apoptosis resistance in infected target cells, are described. These observations, and associated inflammatory cytokine responses, may provide new insights into HFRS and HPS disease pathogenesis. Based on similarities between inflammatory responses in severe hantavirus infections and other hyperinflammatory disease syndromes, we speculate whether some therapeutic interventions that have been successful in the latter conditions may also be applicable in severe hantavirus infections.

## Abstract 2

https://onlinelibrary.wiley.com/doi/full/10.1111/joim.12919 ‐ 15th Key symposium ‐ “Innate immunity”.

## Introduction

**Figure 1:**
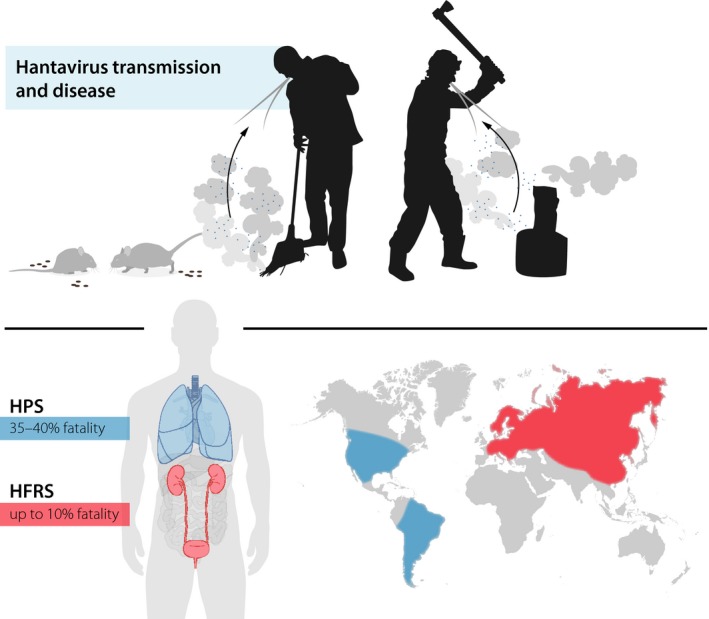
Transmission of pathogenic hantaviruses including Puumala virus (PUUV) to humans occurs predominantly through the inhalation of dust containing virus‐contaminated rodent excreta (illustrated in the upper part of the Figure). In a global perspective, two main hyperinflammatory clinical syndromes can be distinguished following infection with different species of hantaviruses: haemorrhagic fever with renal syndrome (HFRS) and hantavirus pulmonary syndrome (HPS). HFRS is the predominant hantavirus‐induced disease syndrome in Eurasia whilst HPS dominates in the Americas. Many aspects of HFRS and HPS are shared between the two diseases, and the pathogenesis is likely similar even if there are some differences in organ manifestations and, importantly, in severity (illustrated in the lower part of the Figure). In the present review, we discuss recent insights into the innate and adaptive cell‐mediated immune responses to human PUUV infection.

Hantaviruses (also referred to as orthohantaviruses) are zoonotic viruses that belong to the *Bunyavirales* order. The distribution of different hantavirus strains depends on the geographic location of each strains’ specific natural host 1. Transmission of pathogenic hantaviruses to humans occurs predominantly through the inhalation of dust from virus‐contaminated rodent excreta (Fig. 1). In infected humans, hantaviruses mainly target vascular endothelial cells, but they also infect epithelial cells, mononuclear phagocytes (MNP), follicular dendritic cells (DC) and likely also other types of cells 2, 3, 4, 5. Although hantaviruses affect several cellular functions, infection with hantaviruses is not cytopathic *per se* 6, 7.

In a global perspective, hantaviruses cause two related hyperinflammatory syndromes: haemorrhagic fever with renal syndrome (HFRS), mainly caused by the Hantaan, Seoul, Dobrava and Puumala (PUUV) viruses; and hantavirus pulmonary syndrome (HPS), mainly caused by the Andes and Sin Nombre viruses. HFRS is the primary hantavirus‐induced disease syndrome in Eurasia whilst HPS dominates in the Americas 8. Many aspects of HFRS and HPS are shared between the two diseases, and the pathogenesis is likely similar even if there are some differences in organ manifestations and, importantly, in severity. Infection leads to an excessive immune activation including massive cytokine responses and activation of cytotoxic lymphocytes 9, 10, 11, 12, 13, 14. Patients also show increased infiltration of immune cells in organs 13, 15, 16, 17, 18. Together, these responses likely contribute to the pathological responses observed following infection. In more detail, early disease manifests with flu‐like symptoms and affection of specific organs and later on, in severe cases, symptoms such as hypotension, acute shock, vascular leakage, kidney failure and lung failure 1, 2, 4, 19. Reported case‐fatality rates are up to 10% for HFRS and around 35%–40% for HPS 1, 2, 19 (Fig. 1). There is no specific curative treatment or FDA‐approved preventive vaccine for either HFRS or HPS.

The most common causative agent of HFRS in Europe is PUUV, carried by the bank vole (*Clethrionomys glareolus*) 19. PUUV is widespread across large parts of the continent and causes regular outbreaks when the bank vole population peaks 20. Annually, more than 10 000 individuals are diagnosed with HFRS and numbers are increasing 19. This increase may relate in part to increased awareness by the medical community and to changes in environmental factors including climate change. Furthermore, high seroprevalence has been observed in certain areas of Europe, including in areas where only few cases of HFRS are being diagnosed 19, 21. Whilst PUUV‐associated morbidities are significant, mortality rates are normally low (<1%) with some exceptions in the elderly populations where rates are higher 22.

In this review, we discuss recent insights into the cell‐mediated immune responses generated in response to acute PUUV infection. In this context, we review recent results on MNP, granulocyte, natural killer (NK) cell, as well as CD8 and CD4 T‐cell responses. Still lacking are more comprehensive studies with respect to responses of B cells (apart from serology), unconventional T cells such as mucosal‐associated invariant T (MAIT) cells and γδ T cells, and innate lymphoid cells (ILCs) in acute PUUV infection. Results reviewed here are largely, but not exclusively, based on clinical material collected from hospitalized HFRS patients in Northern Sweden, a highly PUUV endemic area 19. Additionally, we also provide insights into pro‐inflammatory cytokines in PUUV infection and infections caused by other hantaviruses. We then provide novel insights into findings demonstrating the ability of hantaviruses, including PUUV, to cause apoptosis resistance in infected target cells. We discuss briefly how PUUV infection may contribute not only to direct infectious disease‐related pathogenesis but also to other co‐morbidities affecting several organ systems including an increased relative risk of lymphoma. Finally, we discuss possible new treatment strategies for the most severe forms of human hantavirus infection, based on novel findings reviewed here.

## Mononuclear phagocyte responses to acute human PUUV infection

Mononuclear phagocytes, consisting of monocytes, macrophages and DCs, present viral antigens to T cells and produce type I interferons (IFN) and various other cytokines. By this means, they are able to initiate and regulate virus‐specific immune responses 23, 24, potentially also in the course of human hantavirus infections 18, 25. Monocyte‐derived cells and DCs are present in respiratory compartments of humans 26, 27, 28, 29, 30. Since hantaviruses are mainly transmitted via inhalation, studying immunological responses in these compartments is of particular interest. Furthermore, pulmonary dysfunction, a hallmark of HPS, has also been reported in HFRS 31, 32, 33. Based on these reports, we set out initially to study infiltration of MNPs in the airways of PUUV‐infected patients.

### Infiltration of MNPs in the airways

**Figure 2:**
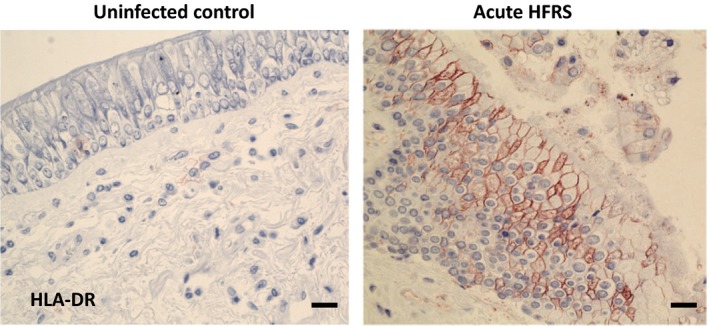
Infiltration of antigen‐presenting cells and other immune cells in the airways during acute Puumala virus‐caused HFRS. Shown are representative images of HLA‐DR staining in endobronchial biopsies in acute HFRS. Specific staining appears in red, and cell nuclei are counterstained with haematoxylin in blue 17. Visualization was performed using immunohistochemistry. Scale bar, 50 μm.

Large numbers of HLA‐DR‐positive MNPs were observed in endobronchial biopsies taken from HFRS patients early after onset of disease 17. Compared to uninfected controls, HFRS patients showed significantly increased numbers of CD11c^+^ cells in the lamina propria and epithelium. In addition, cells expressing the plasmacytoid DC (pDC) marker CD123 were also elevated in the lamina propria. Further analysis revealed a positive association between levels of CD8^+^ cells and CD11c^+^ cells 17. Taken together, these results indicate an infiltration of several MNP subpopulations into the airways during HFRS, coinciding with increased levels of CD8 T cells (Fig. 2).

### Reduced numbers of MNPs in peripheral blood

Since monocytes often participate in the inflammatory response following viral infection 23, we hypothesized that in peripheral blood, the number of monocytes, as reported for other acute viral diseases 34, 35, 36, would also increase in PUUV‐infected patients. The frequencies of classical (CD14^+^CD16^−^), intermediate (CD14^+^CD16^+^) and nonclassical (CD14^−^CD16^+^) peripheral blood monocytes were hence analysed. Surprisingly, we observed that the absolute number of all these subsets of monocytes decreased in peripheral blood during acute HFRS. The reduction in cell numbers coincided with high viral load in the patients. At convalescence, when no virus was detected in plasma, the monocyte numbers were normalized 17.

The loss of monocytes in circulation during acute HFRS led to studies of DCs, important determinants of viral disease outcome 37. Focus was first directed to the two myeloid DC (mDC) subsets found in peripheral blood: conventional (c)DC1 (CD141^+^ mDCs) and cDC2 (CD1c^+^ mDCs). A dramatic reduction in both cDC subsets was observed during acute infection. Subsequently, levels of cDC1s and cDC2s normalized 17. As observed for monocytes, the reduction in cDC1s and cDC2s coincided with high viral load in the patients.

During acute infections, pDCs normally produce high levels of type I IFNs. Yet, levels of IFN‐α might not be elevated in blood during the acute phase of HFRS 38. Numbers of blood pDCs, as defined by CD123 and CD303 expression, were also significantly reduced during acute HFRS. Later on, blood pDC levels returned to normal levels 17. Similar to what was observed for mDCs, the loss of blood pDCs also coincided with high viral load.

### Upregulation of CCR7 on peripheral blood MNPs

Hantaviruses are not cytopathogenic (39; see also further below in this review), indicating that the loss of monocytes and DCs from peripheral blood could be due to redistribution of these cells to other sites, including the airways. To identify cellular indicators of trafficking from blood to tissues 40, 41, the level of the chemokine receptor CCR7 was assessed on the surface of MNPs remaining in the peripheral blood 17. Normally, only few such cells express CCR7. However, in the patients, a subset of these MNPs expressed CCR7. cDC2s also upregulated CCR7 expression, whilst no or only very low levels of CCR7 expression were observed on cDC1s 17. Compared to CCR7^−^ cells, CCR7^+^ cells showed higher CD70 expression (classical monocytes and cDC2s) and CD86 expression (cDC2s), suggesting a more mature phenotype 17. Taken together, the results suggested that monocyte and DC numbers decreased in circulation during acute PUUV infection, and that the remaining MNPs expressed higher levels of migratory receptors such as CCR7, facilitating migration from the blood.

## Granulocyte responses to acute human PUUV infection

Neutrophils, an essential part of the innate immune system, are short‐lived cells. They are the most common type of white blood cell and migrate towards sites of inflammation, helping to resolve infection. Higher levels of peripheral blood neutrophils have been observed in PUUV‐infected patients with moderate to severe symptoms than in patients with mild symptoms 42. Further, PUUV‐infected patients show increased levels of neutrophil activation products 43. Hantavirus infection has also been shown to induce neutrophil extracellular trap formation, though the mechanism behind this finding is debated 43, 44. These observations, along with findings showing that HFRS patients display elevated systemic levels of histone‐double‐stranded DNA complexes as well as antinuclear antigen antibodies, suggest a potential role for neutrophils in hantavirus‐induced immunopathology 43, 44, 45.

## NK cell responses to acute human PUUV infection

Natural killer (NK) cells play a crucial role in the early defence against viruses. Corroborating this notion, specific NK cell deficiencies often predisposes for life‐threatening virus infections 46, 47. NK cells can eliminate virus‐infected cells, for example via the cytotoxic granule‐dependent pathway, and produce antiviral IFN‐γ and pro‐inflammatory cytokines such as TNF 46. NK cells can also be activated by cytokines induced by virus infection 48, such as type I IFNs, IL‐12, IL‐15, IL‐18 and IL‐21 49, 50.

### NK cells rapidly expand and persist at elevated levels

**Figure 3:**
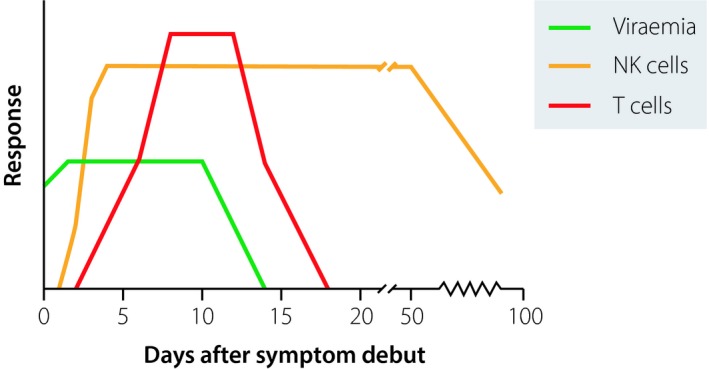
NK cell and T‐cell immune responses observed following Puumala virus (PUUV) infection. Generally, in viral infections, innate immune responses peak after a few days. In contrast, in a cohort of hospitalized PUUV‐infected individuals, a strong NK cell response was elicited and cell numbers remained elevated over several weeks well beyond the resolution of viraemia. The patients also displayed a vigorous, albeit transient, CD8 T‐cell response largely coinciding with viraemia.

In a cohort of PUUV‐infected patients, we initially set out to assess absolute numbers of total lymphocytes, total NK cells and distinct NK cell subsets in peripheral blood. Early after onset of symptoms, NK cell numbers were found to be low in peripheral blood, as has also been observed by others 18. The initial reduction in peripheral blood NK cells is possibly caused by extravasation of NK cells into tissues 51, 52. After this initial drop, CD56^dim^ NK cell numbers (the main population of NK cells in peripheral blood 53, 54) increased markedly and peaked approximately 10 days following onset of clinical symptoms 10, 15. Surprisingly, in a majority of the patients, CD56^dim^ NK cell numbers remained elevated for at least 2 months. At 15 months after infection, after full resolution of clinical symptoms, CD56^dim^ NK cell numbers were normalized 10 (Fig. 3).

Significantly elevated levels of CD69 were observed on CD56^dim^ NK cells during the acute phase of disease 55. Levels of CD69^+^ CD56^dim^ NK cells subsequently decreased over time. During the acute phase, CD56^dim^ NK cells also expressed significantly elevated levels of NKG2D and 2B4 (activating NK cell receptors), NKp30 and NKp46 (natural cytotoxicity receptors), as well as granzyme B and perforin 55. Taken together, this shows that cytotoxic CD56^dim^ NK cells are highly activated during PUUV infection.

### Increased NK cell numbers are a result of induced proliferation

To explore whether the increased numbers of activated CD56^dim^ NK cells were a consequence of proliferation, levels of the proliferation marker Ki67 were assessed in NK cells from PUUV‐infected HFRS patients. Strikingly, Ki67 expression was detected in up to half of the CD56^dim^ NK cells during the first 10 days after onset of HFRS 10. Thereafter, the level of Ki67‐expressing CD56^dim^ NK cells decreased.

Certain cytokines are known to trigger NK cell proliferation and persistence. Interestingly, we observed elevated plasma levels of IL‐15, but not of IL‐2 or IL‐12, up to 60 days after onset of disease 10, suggesting that IL‐15 might be involved in the observed CD56^dim^ NK cell proliferative responses. Corroborating this notion, *in vitro*, hantavirus infection was found to induce transcription of IL‐15 and IL‐15Rα mRNAs and cause increased IL‐15 and IL‐15Rα cell surface expression 55. Upon neutralization of IL‐15, fewer NK cells expressed CD69 55, suggesting that trans‐presented IL‐15 may induce activation of CD56^dim^ NK cell upon contact with PUUV‐infected cells.

Noteworthy, besides contributing to NK cell proliferation, IL‐15 can also promote the survival of proliferating NK cells 56. This may, at least in part, occur via IL‐15‐induced production of the anti‐apoptotic protein Bcl‐2 57. In this respect, compared with nonproliferating (Ki67^−^) NK cells, proliferating (Ki67^+^) NK cells were found to express elevated Bcl‐2 levels 10, which may inhibit apoptosis in proliferating NK cells, leading to an accumulation of these cells.

### Hantavirus‐infected endothelial cells upregulate the NKG2C ligand HLA‐E

Specific NK cell‐activating receptors play important roles in stimulating proliferation and in control of viral infections. A common feature of these receptors is that they can recognize virus‐associated and/or virus‐induced cellular proteins on infected cells 58. To identify possible NK cell‐activating receptors that might be involved in the recognition of target cells, primary human endothelial cells were infected. Hantavirus‐induced changes in NK cell receptor‐ligand expression were then analysed. Increased levels of intercellular adhesion molecule 1 (ICAM‐1), a ligand for lymphocyte function‐associated antigen 1 (LFA‐1), were observed on the cell surface of hantavirus‐infected cells 10. ICAM‐1–LFA‐1 interactions play important roles for NK cells, including promoting lymphocyte adhesion to endothelial cells, NK cell activation, and polarization of NK cell lytic granule release 59. Furthermore, HLA‐E was found to be significantly upregulated. Importantly, HLA‐E is a ligand for the activating NK cell receptor NKG2C 60 implicated in cytomegalovirus (CMV) infection 61, 62, 63.

### Expanding and persisting NK cells are confined to an NKG2C‐expressing subset

The latter observations led us to address whether NKG2C might be involved in the NK cell response to hantavirus infection. In this respect, during the acute phase, HFRS patients showed significantly higher levels of NKG2C^+^ NK cells compared with uninfected controls 10. Furthermore, NKG2C^+^ NK cell expansion, and persistence, was observed in the patients. This expansion was specific for NKG2C^+^ cells and accounted for a large part of the overall CD56^dim^ NK cell expansion in the patients 10. Phenotypically, a large proportion of these NKG2C^+^ cells expressed CD57 and inhibitory killer cell immunoglobulin‐like receptors (KIRs) after expansion, suggesting that they represent highly mature and terminally differentiated CD56^dim^ NK cells 54.

Skewing of the NK cell repertoire towards dominance of certain subsets with narrow phenotype properties, such as the expanded NKG2C^+^ NK cells, leads to an overall lower diversity within the NK cell compartment 64. Noteworthy, a similar disturbance of the NK cell receptor repertoire occurs during chronic hepatitis C virus (HCV) infection and appears to persist for years after clearance of HCV 65. Potential long‐term consequences of such skewing, be it increased susceptibility to subsequent infections and/or cancer, still need to be addressed in future studies.

### CMV infection and NKG2C^+^ NK cell expansion in hantavirus infection

Although degrees of infection vary between different geographical regions around the world, most humans are infected with CMV. CMV seropositivity has been associated with increased frequencies of peripheral blood NKG2C^+^ NK cells 61, 62. In this context, three out of the 16 PUUV‐infected patients included in the NK cell cohort study reviewed above were CMV IgG^−^ 10. Interestingly, at the acute phase of disease, these three patients had lower absolute numbers of NKG2C^+^ NK cells than the majority of CMV IgG^+^ patients and displayed no subsequent NKG2C^+^ NK cell expansion. Based on these observations, a previous CMV infection may have primed a NKG2C^+^ NK cell population for expansion.

## T‐cell responses to acute human PUUV infection

T cells are believed to contribute to hantavirus pathogenesis 11, 66, 67. High frequencies of hantavirus‐specific memory CD8 T cells have been observed years after patients recovered from their infection 12, 68. However, the primary antiviral CD8 Tcell response, including formation of antigen‐specific T cells and Tcell memory 69, 70, has not been extensively characterized in human hantavirus infection.

### PUUV‐infected patients display increased levels of airway CD8 T cells

To investigate local T cell responses, endobronchial biopsies and bronchoalveolar lavage (BAL) were sampled from PUUV‐infected HFRS patients, and then, CD8 T cells were analysed 15. Compared with control subjects, HFRS patients showed increased numbers of CD8 T cells in the epithelium and increased numbers of submucosal CD4 T cells and CD8 T cells. Analysis of BAL fluid showed higher proportions of CD8 T cells and NK cells in the patients. T cells showed signs of activation as deduced by observations of elevated HLA‐DR and CD25 expression 15. In relation to these studies, the magnitude of pulmonary cytotoxic lymphocyte responses has been shown to correlate with the severity and systemic organ dysfunction, including vascular leakage, hypotension, cardiac dysfunction, supplemental oxygen treatment, renal failure and cell damage 71. Taken together, this indicates an increased lung Tcell response of patients infected with PUUV.

### Identification of responding CD8 T cells in peripheral blood

Parallel to these studies, a detailed characterization of peripheral blood T cells was carried out in acute PUUV infection 9. Studies of cells from peripheral blood allowed for a thorough analysis of the temporal dynamics of the Tcell response, including in‐depth studies of activation status and effector cell phenotypes. Early after infection, relative levels of CD8 T cells were increased compared to levels of CD4 T cells. At day 60 after infection (convalescent phase), CD8 Tcell levels were normalized to those observed in uninfected individuals. During the acute phase of infection, a substantial proportion of CD8 T cells were found to express Ki67, CD38 and HLA‐DR. The Ki67^+^CD38^+^HLA‐DR^+^ CD8 Tcell subset peaked at day 6 and then decreased by day 10, to be virtually undetectable at day 60 9. The decrease in levels of effector CD8 T cells coincided with decreased viral load in the patients 9 (Fig. 3). Levels of Ki67^+^CD38^+^HLA‐DR^+^ CD4 T cells were not elevated early after symptom debut. However, increased CD4 Tcell Ki67 expression, and a trend towards higher CD38 expression, was observed 9.

### Responding CD8 T cells display an effector phenotype

To analyse whether the Ki67^+^CD38^+^HLA‐DR^+^ CD8 T cells represented effector CD8 T cells, the expression patterns of CCR7, CD28, CD45RA, CD127, granzyme B and perforin were analysed on responding (Ki67^+^) and nonresponding (Ki67^−^) CD8 T cells 9. Responding CD8 T cells consistently expressed low levels of CCR7, CD45RA and CD127, whilst the co‐stimulatory molecule CD28 was expressed on approximately half of the Ki67^+^ CD8 T cells. Of the Ki67^+^ CD8 T cells, more than 70% were CCR7^−^CD45RA^−^CD127^−^CD28^+/−^ at day 6 after symptom debut. This phenotype differed from that of Ki67^−^CD8 T cells at the same time‐point as well as from uninfected controls. Furthermore, CD8 T cells showed a high frequency of perforin and granzyme B expression during the acute phase, which had normalized at day 60 9. These data suggest that Ki67^+^CD38^+^HLA‐DR^+^ CD8 T cells make up the effector cell CD8 Tcell population that respond to PUUV infection.

### No expansion of regulatory CD4 T cells

A few studies exist with respect to CD4 T cells, including regulatory FoxP3^+^ CD4 T cells, in clinical hantavirus infection 72, 73, 74, 75. To more directly identify mechanisms that could balance the effector Tcell response, we analysed FoxP3^+^CD25^high^CD127^low^ T regulatory (T_reg_) cells in PUUV‐infected patients 9. To summarize studies performed, CD4 T cells with a regulatory phenotype are present in peripheral blood during acute hantavirus infection but seem not to be increased in frequency.

### Transient expression of inhibitory receptors on responding T cells

Expression of inhibitory receptors is a mechanism to ensure Tcell tolerance during steady‐state conditions. These receptors regulate Tcell responses and have been linked to Tcell dysfunction during chronic viral infection 76. However, they have not been extensively analysed during the early phase of acute human viral infection. In this context, expression of PD‐1 and CTLA‐4 on responding T cells was analysed during acute PUUV infection 9. PD‐1 was not detected on CD8 T cells from patients in the present cohort 9. In contrast, CTLA‐4 was detected on 40% of the CD8 T cells during the acute phase but then rapidly declined. At day 60, almost no CD8 T cells expressed CTLA‐4.

The analysis was also extended to CD4 T cells. CTLA‐4 was detected on many CD4 T cells early after symptom debut. Additionally, PD‐1 was expressed on 15% of CD4 T cells. Noteworthy, almost all of the PD‐1‐expressing CD4 T cells expressed CTLA‐4. CTLA‐4 and PD‐1 expression on CD4 T cells then decreased after the early acute phase 9. Taken together, this suggests that cell‐intrinsic processes may balance effector T‐cell responses early after onset of disease.

## Hantavirus‐infected cells are protected from cytotoxic lymphocyte‐mediated apoptosis

In the previous sections, we have described how an acute PUUV virus infection generates a high number of activated NK cells that can persist for months 10. In parallel, strong CD8 Tcell responses are observed during the acute phase 9. However, no obvious damage of infected endothelial cells has been observed in autopsies from deceased patients 4, 5, 6. This suggested to us that hantaviruses, including PUUV, might protect infected cells from being killed by cytotoxic lymphocytes.

To study the effects of cytotoxic lymphocytes on hantavirus‐infected cells *in vitro*, we exposed infected primary endothelial cells to activated NK cells. Levels of NK cell degranulation did not differ between NK cells co‐incubated with infected compared to uninfected cells. Strikingly, however, whilst the NK cells readily killed uninfected cells, infected cells were not killed 39, 77. Interestingly, we observed that the hantavirus nucleocapsid protein interfered with granzyme B and caspase 3 enzymatic activities 39, 77. This suggests that hantavirus inhibits cytotoxic granule‐mediated apoptosis induction, hence protecting infected cells from being killed by cytotoxic lymphocytes.

## Acute and possible long‐term effects of clinical PUUV and other hantavirus infections

Reports on the clinical manifestations of HFRS have largely focused on acute renal failure. Up to 5% of PUUV and 50% of Dobrava virus‐infected hospitalized HFRS patients have been reported to require dialysis during the acute stage of disease 19. However, several extrarenal manifestations are also observed in HFRS including cardiac, pulmonary, ocular and hormonal disorders 19. In a Korean study, extrarenal manifestations involving major organs were reported to occur in one‐third of HFRS patients 78. This included cardiovascular, central nervous system, pancreatobiliary symptoms, and major bleedings 78. A Finnish prospective study of cardiac dysfunction in hospital‐treated HFRS patients using electrocardiogram (ECG) and electrocardiography as well as serial measurement of cardiac troponins showed that cardiac involvement is common 79. In acute‐phase ECG, changes were observed in more than half of the HFRS patients and impairments of cardiac contractions and pericardial effusions were reported in some patients. Aberrances were usually normalized after 3 months 79. A Swedish study addressing the causes of death during and after HFRS concluded that cardiovascular disorders were the cause of death in more than half of patients who died in Sweden during the first year after HFRS 80. Corroborating this study, a related report demonstrated a significantly increased risk of acute myocardial infarction and stroke during HFRS 81. More recently, risk of thromboembolism following HFRS has also been addressed where a significantly increased risk of venous thromboembolism was observed early after on onset of HFRS 82.

Given the observations on hantavirus‐mediated inhibition of apoptosis, and the notion that apoptosis resistance is one of the hallmarks of cancer 83, we addressed whether PUUV infection might be linked to cancer. This study was done by cross‐running the Swedish HFRS register with the Swedish Cancer Registry. Over 6500 HFRS diagnosed individuals in Sweden were included in the study. Of these patients, more than 350 were diagnosed with cancer. Strikingly, amongst these patients, the relative risk of developing lymphoma was significantly increased 84. The highest risk of lymphoma was observed early after HFRS and then decreased with time. The possibility of a causal link between PUUV and lymphoma development remains to be investigated.

## Inflammatory responses in PUUV and other hantavirus infections

Despite recent efforts to characterize the human immune response to hantavirus infection, mechanisms behind the pathogenesis of HFRS and HPS remain unknown. Not unlikely, the virus‐induced immune responses may contribute to the pathology of the diseases 1, 19. It has been known for long that human hantavirus infection induces a strong inflammatory response with increased levels of pro‐inflammatory cytokines 85, 86, 87, 88, 89. How these responses might affect the disease outcome has, however, not been studied in detail.

In 1996, elevated plasma levels of IL‐6, IL‐10 and TNF were reported in PUUV‐infected HFRS patients 90. Since then, several reports have confirmed findings of strong cytokine responses in PUUV‐infected patients, including elevated plasma levels of IL‐2, IL‐6, IL‐8, IL‐10, TNF, TGF‐β1, IFN‐γ, VEGF and other inflammatory markers such as CRP 88, 89, 91, 92, 93, 94, 95. Elevated IL‐6 levels in plasma have been associated with more severe HFRS 89. Elevated levels of IL‐6 and other cytokines such as IL‐8, IL‐10, TNF and IFN‐γ have also been detected in Dobrava virus‐infected and Hantaan virus‐infected HFRS patients 96, 97, 98, 99 and in HPS patients 87, 100, 101 indicating that elevated levels of these cytokines are a general consequence of human hantavirus infection.

In an attempt to more directly address correlates with disease outcome and severity during hantavirus infections, we recently characterized the systemic inflammatory response in one of the largest cohorts of HPS patients analysed to date, including 93 Andes virus‐infected HPS patients out of whom 34 had a fatal outcome 102. The results showed that not only were inflammatory markers highly increased, but also markers of microbial translocation and intestinal damage. Interestingly, by multivariate analyses, it was shown that intestinal fatty acid‐binding protein (I‐FABP), a marker of intestinal injury, was independently associated with increased odds of a fatal outcome. When comparing fatal and nonfatal cases in univariate analyses, IL‐6 and IL‐15 were the only two cytokines associated both with increased odds of severe disease and fatal outcome. Interestingly, multivariate analyses identified IL‐6 as an independent marker of severe disease, suggesting that this cytokine may have an important role in HPS pathogenesis 102.

## Novel possible treatment options for patients with severe hantavirus infection

As mentioned above, hyperinflammation is a hallmark of severe hantavirus infection 1. In the most severe hantavirus infections, as seen primarily in the development of HPS following infection with the Sin Nombre or Andes viruses, but also in some HFRS cases, inflammatory responses coincide with pulmonary vascular leakage, resulting in high mortality rates due to fulminant hypoxic respiratory failure and/or cardiogenic shock. In these situations, extracorporeal membrane oxygenation (ECMO) represent a possibly life‐saving intervention 103, 104. Notably, treatment with corticosteroids or antiviral drugs has not been successful 105. Hence, novel treatment strategies are needed for the most severe cases of hantavirus infection. We here discuss some possible alternative strategies, based in part on results discussed above.

The first strategy emerges from patients with familial haemophagocytic lymphohistiocytosis (FHL). FHL is a hyperinflammatory, often life‐threatening, primary immunodeficiency syndrome affecting infants or young children 106. The disease is related to mutations in the perforin‐encoding gene or in genes encoding proteins required for the exocytosis of perforin‐ and granzyme‐containing cytolytic granules. Because of these mutations, cytotoxic lymphocytes of the patients cannot eliminate virus‐infected cells via the cytolytic pathway 107. The described mutations are strongly associated with the development of a hyperinflammatory syndrome that shares many similarities to symptoms observed in severe hantavirus infections 108. In a historic context, patients with FHL had a dismal prognosis. However, since the implementation of immunochemotherapy that serves to control excessive immune activation, one has efficiently been able to resolve the oftenfatal hyperinflammatory condition 109. In view of these accomplishments, one may speculate whether some aspects of this radical immunochemotherapy could be used in the most severe cases of hantavirus‐induced hyperinflammatory conditions.

The second strategy emerges from cancer immunotherapy, a field that has undergone a remarkable progress in recent years. In this context, recently developed chimeric antigen receptor (CAR) T cells and immune checkpoint inhibitors have shown remarkable efficiency in clinical trials 110, 111. However, treatment with CAR‐T cells, in particularly, has been associated with some potentially fatal adverse effects. The latter include hyperinflammatory conditions 112. The pathogeneses of these ‘cytokine release syndromes’ (CRS) are not completely understood, but it likely involves several types of immune cells producing IL‐6 and other pro‐inflammatory cytokines. This has led to the introduction of successful anti‐IL‐6R treatment of severe CRS 113. In relation to recent findings from our own laboratory 102, and other laboratories 89, 100, increased levels of IL‐6 have also been observed and found to be associated with severe disease in hantavirus infection caused by Andes and Puumala viruses. This finding suggests the possibility of trying similar anti‐IL‐6R therapeutic strategies for treatment in the most severe cases of hantavirus infection.

Finally, it is not clear whether and to what extent the severe NK cell activation contributes to disease pathology in hantavirus infection. If it does, one may consider strategies to dampen this activation. We earlier described how the cytokine IL‐15 contributed to driving the massive NK cell response following interaction with hantavirus‐infected cells and levels of IL‐15 have also been associated with severe disease and fatal outcome. IL‐15 is thought to drive immunopathology in certain other clinical situations, conditions that might be reversed by blocking anti‐IL‐15 antibodies 114, 115, 116. Thus, IL‐15 might represent a possible therapeutic target for severe hantavirus infection.

**Figure 4:**
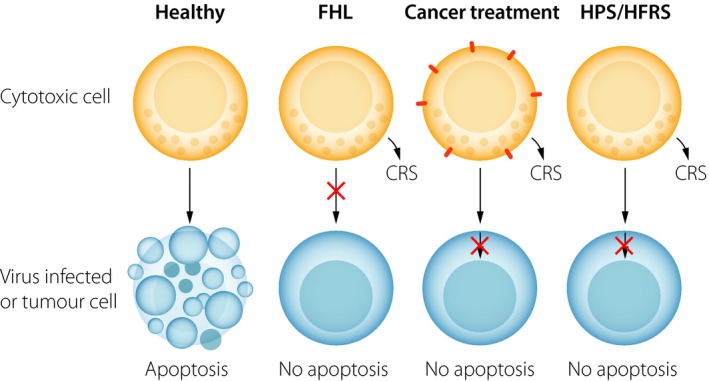
Similarities between inflammatory responses in severe hantavirus infections and other, nonrelated, hyperinflammatory disease syndromes. Cytotoxic cells of the immune system, such as natural killer (NK) cells and cytotoxic T cells (CTL), normally possess the capacity to kill virus‐infected cells and tumour cells (panel 1). Killing does however not always occur. In rare cases, such as in familial lymphohistiocytosis (FHL), mutations in genes affecting production of proteins such as perforin or proteins affecting degranulation lead to an impaired elimination of target cells (panel 2). In other cases, such as cancer, CTL cells may encounter apoptosis‐resistant tumour cells (panel 3). Finally, in the case of hantavirus infection, including Puumala virus infection, cytotoxic cells likewise encounter apoptosis‐resistant cells (panel 4). Interestingly, all displayed conditions above (illustrated in panels 2–4) are associated with severe inflammation or ‘cytokine release syndromes’ (CRS). Handling of these often‐severe clinical conditions differs. In the case of reducing CRS in FHL (panel 2), immunochemotherapy (dexamethasone and etoposide) has been successfully implemented as a treatment strategy. In the case of reducing CRS following adoptive CAR‐T cell treatment (panel 3), treatment interfering with IL‐6 signalling has proven efficient. The question is whether any of the latter two treatment strategies could find a role in severe clinical hantavirus infection (panel 4).

We cannot say whether any of the discussed treatment options outlined here would be effective or even at all possible. It is, however, noteworthy that HFRS/HPS, FHL and cancer have a common denominator in terms of pathogenesis, the inability of the immune system to efficiently eliminate target cells albeit for different reasons (Fig. 4). We speculate that, in all three types of diseases, this could contribute to hyperinflammatory responses/CRS. In this context, lessons learnt from the latter two conditions (FHL and side effects of CAR T cell treatment during cancer) may serve to improve the clinical outcomes of patients with severe forms of clinical hantavirus infection, critical conditions associated with rapid progress and high fatality rates. More research is clearly needed in this field.

## Conflict of interest statement

No one of the authors have any conflict of interest in relation to the work reviewed in this paper.
